# P-2155. Early Diagnosis of Dengue, Malaria, Scrub Typhus, Leptospira Using CBC and LFT Parameters in a Tertiary Care Hospital - A 10-year retrospective study

**DOI:** 10.1093/ofid/ofae631.2309

**Published:** 2025-01-29

**Authors:** Suresh Kumar, S Shibi, R Sanjai, S Shobana

**Affiliations:** Apollo Speciality Hospitals, Chennai, Tamil Nadu, India; Apollo Hospital, Chennai, Tamil Nadu, India; Apollo Hospital, Chennai, Tamil Nadu, India; Apollo Hospital, Chennai, Tamil Nadu, India

## Abstract

**Background:**

Dengue, malaria and scrub typhus/leptospirosis are significant infectious diseases prevalent in tropical and subtropical regions. Timely and accurate diagnosis of these diseases is crucial for initiating appropriate treatment and preventing complications. Dengue shows increased Hb, AST should be greater than ALT, as well as a decrease in platelets, along with lymphocytes in the differential count should be observed. Scrub typhus/leptospirosis reveal dropping platelets, along with an increase in total count, alkaline phosphate, and GGTP should be observed. Malaria indicates reduced Hb and platelets, along with an increase in total bilirubin, alkaline phosphate, and GGTP should be noted. Additionally, monocytes and lymphocytes should be seen in the differential count.

**Methods:**

This retrospective study conducted between January 2014-March 2024 at a tertiary hospital in India. We systematically reviewed the medical records of these patients, focusing on demographical, CBC and LFT parameters. Data were entered into an Excel spreadsheet, and descriptive statistics were employed for data interpretation.

**Results:**

During the study period, a total of 99,431 were admitted. For Dengue 80.93% CBC and LFT were followed. In Scrub Typhus/Leptospirosis 58.6% were followed and in malaria 62.5% followed the CBC and LFT as shown in Table 1.

**Table 1:** Cumulative summary of Dengue, Malaria, Scrub Typhus/Leptospira

CATEGORY	FREQUENCY (N)	PERCENTAGE (%)
Total number of patients admitted	99,431	100%
Total number of Dengue test done	12,020	12.08%
NS-1 Ag Positive	1,726	14.35%
CBC and LFT matched	1,397	80.93%
Not matched	329	19.06%
Total number of Scrub Typhus/Leptospirosis done	2,825	2.84%
Positive IgM ELISA	150	5.30%
CBC and LFT matched	88	58.6%
Not matched	62	41.3%
Total number of Malaria test done	7,880	7.92%
Positive MP by QBC	32	0.40%
CBC and LFT matched	20	62.5%
Not matched	12	37.5%

**Conclusion:**

This study highlights the significant burden of Dengue, Scrub Typhus/Leptospirosis, and Malaria in a tertiary care setting in India. While Dengue was the most prevalent, all three diseases demonstrated the potential for early diagnosis by utilizing both CBC and LFT parameters.

**Disclosures:**

All Authors: No reported disclosures

